# Apelin Effects Migration and Invasion Abilities of Colon Cancer Cells

**DOI:** 10.3390/cells7080113

**Published:** 2018-08-20

**Authors:** Marta Podgórska, Katarzyna Pietraszek-Gremplewicz, Dorota Nowak

**Affiliations:** Department of Cell Pathology, Faculty of Biotechnology, University of Wroclaw, Joliot-Curie 14a, 50-383 Wroclaw, Poland; marta.wysocka@uwr.edu.pl (M.P.); katarzyna.pietraszek-gremplewicz@uwr.edu.pl (K.P.-G.)

**Keywords:** apelin, adipokines, apelin receptor, migration, invasion, colon cancer, actin, cofilin, metalloproteases

## Abstract

Colon cancer is one of the most common cancer types. Its positive correlation with general obesity has led to increasing amounts of research focusing on the role of adipokines in colon cancer development. Apelin is a peptide released by adipose tissue that could affect many cellular processes connected with carcinogenesis. In this study, we examined the role of apelin in the motility regulation of colon cancer cells. We showed that the effect of four different apelin peptides increased the ability of cancer cells to migrate and invade examined cells trough influencing migratory protrusions formation and actin cytoskeleton rearrangement. Additionally, using confocal microscopy, we noticed that apelin stimulated the proteolytic activity of cancer cells, especially increasing the level of membrane-type 1 matrix metalloprotease. Taken together, apelin increased the movement of colon cancer cells through several possible mechanisms. Moreover, better understanding the process through which apelin regulates cancer development is still necessary to the creation of novel anti-cancer therapy.

## 1. Introduction

Colon cancer is an aggressive disease that continues to have a considerable impact on global health. According to predictions, the number of colon cancer cases will continue to grow in the next years, especially in the young adult group [1,2]. Moreover, strong evidence shows that colorectal cancer is positively correlated with obesity. There are few plausible mechanisms that explain this correlation, including insulin resistance, chronic inflammation and altered levels of growth factors or adipokines [3]. For this reason, studies examining colon cancer biology and its correlation with obesity, which could help find new and more effective diagnostic and therapeutic targets, are still necessary.

Apelin is a secreted peptide, synthetized as a 77-amino acid precursor called preproapelin, which belongs to the adipokine family. Preproapelin may generate several active fragments: a 36-amino acid peptide corresponding to the sequence 42–77 (apelin-36), a 17-amino acid peptide consistent with the sequence 61–77 (apelin-17) and a 13-amino acid peptide equivalent to the sequence 65–77 (apelin-13), which are the most common forms of apelin [4]. This latter fragment may also undergo pyroglutamylation at the level of its N-terminal glutamine residue, which results in its protection from exopeptidase degradation. This form of apelin—[Pyr1] apelin-13, was identified as the major apelin variant in human plasma [5]. Both apelin (APLN) and its receptor (APLNR, APJ) have been implicated as key regulators of central and peripheral responses to multiple homeostatic perturbations. For a long time, apelin was thought to only a role in the regulation of cardiovascular function, angiogenesis, fluid homeostasis and energy metabolism [6,7]. However, recent findings showed that apelin may be a possibly important proangiogenic factor in cancer [8]. Increasing number amount of data also suggest, that that apelin might be a potential anti-cancer therapeutic target [9]. APLN levels increased in non-small cell lung cancer samples in comparison to normal lung tissue and high apelin levels were related to poor overall patient survival [10]. Higher expression levels of APLN and APJ were also detected in hepatocellular carcinoma tumours [11] and in human colon adenomas and adenocarcinomas in comparison to healthy tissue [12]. Exogenous apelin had anti-apoptotic effects on colon cancer cells [12], whereas in human colon cancer cell lines, this peptide stimulated proliferation through the JAG-1/Notch3 signalling pathway [13].

Cell migration plays a significant role in many physiological and pathological processes, such as embryogenesis, wound healing, angiogenesis and metastasis. One of the main factors involved in the regulation of cell movement is actin cytoskeleton [14]. Actin is a conservative protein, implicated in many different processes, such as muscle contraction, cell motility, adhesion and maintenance of shape [15,16]. The organisation of the actin cytoskeleton is regulated by multiple actin-binding proteins (ABPs), which regulate the dynamic balance between monomeric and polymerized (filamentous) form of actin. This proportion may be disturbed in cancer cells [17]. One of the ABPs is cofilin, which interacts with both monomeric and filamentous actin. This protein affects the dynamic remodelling of actin cytoskeleton and can act in two ways: severing actin filaments and increasing depolymerization speed by augmenting the dissociation of monomers from actin filaments [18]. Some data also indicated that cofilin influenced by apelin could stimulate the migration of lung cancer cells [19].

To allow cancer cell growth and metastasis, it is necessary to reorganize and degrade the barrier constituted by the extracellular matrix (ECM) via appropriate proteases, such as matrix metalloproteases (MMPs) [20]. Some data indicated that apelin stimulated the migration of several types of cancer cells, like human lung adenocarcinoma [19], gastric cancer [21] and oral squamous cell carcinoma [22]. Moreover, apelin can affect the invasion of breast cancer cells by inducing the expression of MMP-1 [23], and in the case of gastric cancer cells, MMP-1 and MMP-9 levels were elevated [21]. However, no data focus on the effect of apelin on MMPs-dependent colon cancer cell migration. Therefore, the aim of our studies was to analyse if apelin is able to regulate the migration and invasion abilities of parental LS180 colon cancer cell line and the EB3, 3LNLN and 5W sublines, which have different migration abilities [24]. Moreover, several possible mechanisms of action were analysed.

## 2. Materials and Methods

### 2.1. Chemicals and Reagents

Antibodies to ezrin and cofilin were from Sigma (St. Louis, MO, USA). Antibodies to GAPDH were from Santa Cruz Biotechnology (Santa Cruz Biotechnology Inc., Santa Cruz, CA, USA). Antibody to MT1-MMP was from Merck Millipore (Darmstadt, Germany). Horseradish peroxidase (HRP)-conjugated secondary antibodies were from Cell Signalling Technology (Danvers, MA, USA). The secondary antibodies Alexa Fluor^®^ 488 goat anti-rabbit immunoglobulin G (IgG), Phalloidin Alexa Fluor^®^ 568 and Hoechst 33342 were from Invitrogen (Carlsbad, CA, USA). [Pyr1] apelin-13 (pQRPRLSHKGPMPF), apelin-13 (QRPRLSHKGPMPF), -17 (KFRRQRPRLSHKGPMPF) and -36 (LVQPRGSRNGPGPWQGGRRKFRRQRPRLSHKGPMPF) were from Bachem (Bubendorf, Switzerland), and ML221 was from Sigma. Matrigel™ was from Corning^®^ (New York, NY, USA).

### 2.2. Cell Culture

The human colorectal carcinoma cell line LS180 was obtained from Deutsche Krebsforschungzentrum, Heidelberg, Germany. The selected cell lines EB3, 3LNLN and 5W were obtained from the Institute of Immunology and Experimental Therapy, Polish Academy of Sciences in Wroclaw, Poland. As previously described, the EB3 cell line was received through in vitro selection of LS180 cells. Cell lines 3LNLN and 5W were obtained by in vivo selection of EB3 cells in mice that metastasized to the lymph nodes and liver, respectively [25]. All cell lines were confirmed by LGC Cell Line Authentication Service. Cells were cultivated in MEM-α medium (Corning^®^) containing 10% FBS (Sigma), 2 mM glutamine (Sigma) and antibiotics (100 U/mL penicillin, 100 μg/mL streptomycin) (Sigma) in tissue culture flasks at 37 °C under humidified atmosphere of 5% CO_2_ and passaged twice a week using 0.25% trypsin/0.05% EDTA solution (IITD PAN, Wroclaw, Poland).

### 2.3. Proliferation Analysis

Twenty-four hours after cell seeding onto 96-well plates (3 × 10^3^ cells), apelin peptides (100 nM) in MEM-α medium were added into the cells and incubated in IncuCyte ZOOM Kinetic Live Cell Imaging System (Essen Biosciences Inc., Ann Arbor, MI, USA) for 72 h. The images were taken every 24 h. The results are presented as a proliferation rate (percent of confluence in given time to percent of confluence in time zero). The experiments were performed three times and each independent experiment consisted of three measurements.

### 2.4. Migration and Invasion Assay

Cell migration and invasion assays were performed using Transwell™ filters (PET membrane with 8 µm pore size) (BD Biosciences, Franklin Lakes, NJ, USA) in 24-well plates. Twenty-four hours after seeding on 6-well plates cells were starved in serum-free MEM-α medium for 6 h. Next, cells were seeded (5 × 10^4^ cells) in serum-free MEM-α supplemented with apelin peptides (100 nM) or ML221 (100 nM) directly onto rehydrated Transwell™ insert for migration assay or coated with Matrigel™ (0.5 mg/mL in serum-free MEM-α medium) for invasion assay. The lower part of the well was filled with MEM-α medium containing 20% foetal bovine serum (FBS) and 5 nM EGF or 50% FBS and 5 nM epidermal growth factor (EGF), used as a chemoattractant for migration and invasion assays, respectively. After 24 h, non-migrating cells were removed, the cells migrated through the filter were fixed with 4% formaldehyde, stained with Hoechst 33342 (Invitrogen) and counted using a fluorescent microscope Olympus IX70 (Olympus, Tokio, Japan). The score of migrated or invaded cells was obtained by counting cells that migrated or invaded through the Transwell™ filters from whole insert area. The results are provided as a relative migration or invasion factor (% of control), where control cells are presented as a 100%. The experiments were performed three times and each independent experiment consisted of three replicates.

### 2.5. Cytosolic Fraction Isolation

The cytosolic fraction of the cells was prepared as described by Malicka-Błaszkiewicz and Roth [26]. Briefly, the cells were grown in tissue culture dishes with apelin peptides (100 nM) or ML221 (100 nM) for 24 h, then were gently washed three times with phosphate buffered saline (PBS), scraped with a tissue culture cell scraper and suspended in freshly made monomeric actin stabilizing buffer A (10 mM Tris-HCl, pH 7.4, 0.1 mM ATP, 1 mM dithiothreitol, 0.1 mM CaCl_2_ and 0.25 M sucrose). Then, the cells were centrifuged in 1000× *g*, 5 min at 4 °C and homogenized in 3 volumes of ice-cold monomeric actin stabilizing buffer A with a Dounce homogenizer on ice. After ultracentrifugation of homogenates in 105,000× *g*, 1 h and 4 °C, supernatants used as a cytosolic fraction were collected and frozen in −80 °C. All experiments were performed in triplicates.

### 2.6. Actin Polymerization State Determination

The actin polymerization state was evaluated by measurement of the inhibition of DNase I bovine pancreas [26]. The concentration of monomeric (G) actin was analysed directly in the cytosolic fraction of the cells. The concentration of total (T) actin was determined in diluted cytosolic fraction with monomeric actin stabilizing buffer A without sucrose. This step was critical for completely depolymerizing filamentous (F) actin. The amount of F-actin was calculated by subtracting the amount of G actin from the total actin (F = T − G). The actin polymerization state was determined as the ratio of F-actin to G-actin (F:G actin). One unit of DNase I inhibitor (actin) was the concentration of actin that decreases the activity of 20 mg of DNase I by 10% under standard assay conditions [26]. The results are presented as units of DNase I inhibitor per mg of a sample protein. The experiments were performed three times.

### 2.7. Cell Extracts Isolation

Twenty-four hours after cell seeding onto 6-well plates apelin peptides (100 nM) or ML221 (100 nM) were added into the cells and incubated in MEM-α medium for the next 24 h. After cells were lysed on ice with RIPA lysis buffer (50 mM Tris-HCl, pH 7.4, 150 mM NaCl, 1% Triton X-100, 1% sodium deoxycholate, 0.1% SDS and 1 mM EDTA), supplemented with protease and phosphatase inhibitors cocktails (Sigma) and collected with a cell scraper. After three frozen-thawed cycles and centrifugation in 12,000× *g*, 10 min, at 4 °C, supernatants were collected and used for further analysis.

### 2.8. Western Blotting Assay

The protein concentration in cell extracts was determined using the Bradford procedure [27]. Samples with identical protein quantity (30 µg) were separated by sodium dodecyl sulphate–polyacrylamide gel (10% acrylamide/bis) electrophoresis (SDS-PAGE) and transferred onto nitrocellulose membranes (GE Healthcare, Chicago, IL, USA). After transfer, total protein analysis was performed using Ponceau S staining. Target proteins were probed with specific antibodies and detected using Western blotting Luminol Reagent (Bio-Rad) under ChemiDoc (Bio-Rad) and analysed with Image Lab software (ver. 6.0, Bio-Rad, Hercules, CA, USA). The experiments were performed three times. Image Lab software was used for densitometry analysis of protein bands.

### 2.9. Fluorescent Staining

Twenty-four hours after cell seeding onto sterile coverslips in 24-well plates, apelin peptides (100 nM) or ML221 (100 nM) were added into the cells for next 24 h. Then, cells were fixed with 4% formaldehyde and permeabilized using 0.1% Triton X-100. After blocking with 1% bovine serum albumin (BSA), cells were incubated with specific primary antibodies overnight at 4 °C. Next, the cells were washed three times in PBS and incubated with Phalloidin Alexa Fluor^®^ 568, Hoechst 33342 and secondary antibodies conjugated with Alexa Fluor^®^ 488 for 1 h in room temperature. After washing with PBS, coverslips were mounted in Dako^®^ cytomatic fluorescent mounting medium (Dako). The localization of proteins was analysed using a confocal laser scanning microscope, Leica SP8, with LasX 3.3.0 software (Leica, Wetzlar, Germany). Images were processed using ImageJ 1.52d software (ImageJ, Bethesda, MD, USA) [28]. Blebs quantification was calculated as a percent of cells forming blebs for 100 cells per condition and presented as a percent of control. The experiments were performed three times. The number of MT1-MMPs positive signals was calculated for 30 cells per condition using ImageJ software and quantitative analysis of focal adhesion protocol [28,29] and presented as a percent of control.

### 2.10. Fluorescent-Substrate Degradation Assay

The experiment was performed using a previously described protocol [30]. Sterile coverslips coated with poly-l-lysine (BD Biosciences) were washed with PBS and incubated with 0.5% glutaraldehyde for 15 min in room temperature. Coverslips were washed with PBS and coated with FITC-conjugated gelatine (Invitrogen) for 10 min. After washing with PBS, coverslips were incubated with sodium borohydride for 1 min and washed again with PBS. To determine the proteolytic activity, cells were seeded onto prepared coverslips (5 × 10^4^ cells) and grown with apelin peptides (100 nM) or ML221 (100 nM) for 24 h. Cells were fixed with 4% formaldehyde and stained with Phalloidin Alexa Fluor^®^ 568 for filamentous actin detection. Images were collected using an Olympus IX70 confocal laser scanning microscope (Olympus, Tokyo, Japan) with FLUOVIEW FV1000 software (Olympus, Tokio, Japan). Images were analysed through quantification of darker areas lacking fluorescence, which reflected increased activity of ECM degrading proteases using ImageJ software [28]. The results were calculated as a percent of cells digesting gelatine and then presented as a percent of control. The experiments were performed three times.

### 2.11. Statistical Analysis

All data are shown as a mean ± standard deviations (SD) and their significance was determined using Student’s *t* test. The significance test was set at *p* < 0.05 (*), *p* < 0.01 (**) or *p* < 0.001 (***).

## 3. Results

### 3.1. Apelin Increases Proliferation, Migration and Invasion of Colon Cancer Cells

**Figure 1:**
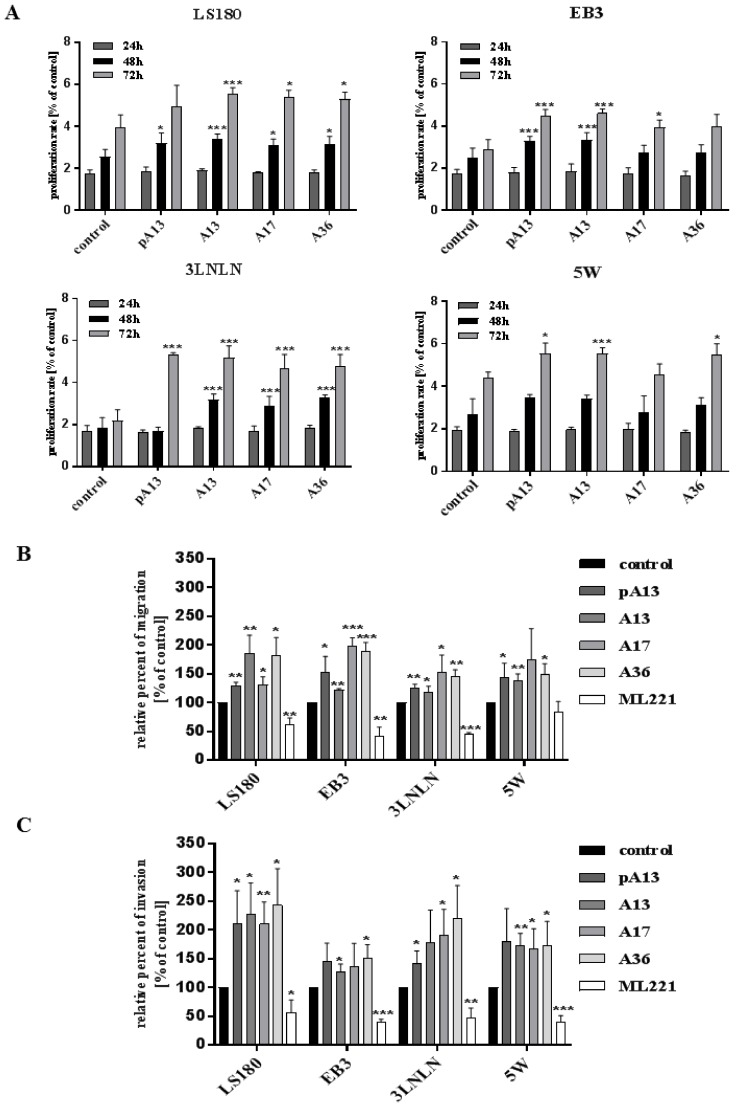
Effect of apelin on proliferation, migration and invasion abilities of colon cancer cells. (**A**) Proliferation rate of colon cancer cells after apelin peptides (100 nM) stimulation. Results are expressed as the mean (proliferation rate) ± SD of three independent experiments. *p* ≤ 0.05 (*), *p* ≤ 0.01 (**), *p* ≤ 0.001 (***); (**B**) Migration of colon cancer cells after apelin peptides (100 nM) or ML221 (100 nM) treatment. Results are expressed as the mean (relative percent of migration) ± SD of three independent experiments. *p* ≤ 0.05 (*), *p* ≤ 0.01 (**), *p* ≤ 0.001 (***); (**C**) Invasion of colon cancer cells after apelin peptides or ML221 treatment. Results are expressed as the mean (relative percent of invasion) ± SD of three independent experiments. *p* ≤ 0.05 (*), *p* ≤ 0.01 (**), *p* ≤ 0.001 (***).

Some studies indicated that apelin could stimulate cell proliferation [23,31]. Thus, we examined the proliferation rate of colon cancer cell lines: parental LS180 and sublines: EB3, 3LNLN and 5W, which were shown to present different migratory potential [24]. Four apelin peptides were tested: [Pyr1] apelin-13 (pA13), apelin-13 (A13), apelin-17 (A17) and apelin-36 (A36). Based on bibliographical data [12] and our studies (Supplementary Figure S1), we selected 100 nM concentration for all apelin peptides, displaying no cytotoxic effect on examined cells, that was able to influence cellular processes. There was no effect of apelin on cell proliferation after 24 h. Therefore, all experiments were performed for that time. Moreover, apelin peptides increased the proliferation rate of tested cells measured for 48 h or 72 h (Figure 1A). Apelin could be also involved in cancer cell migration [19,21,22]. Therefore, we examined migration abilities of colon cancer cell lines. We tested four apelin peptides, as well as the APJ receptor antagonist ML221. In Transwell™ migration assay apelin peptides stimulated cell motility in all cell lines, whereas ML221 decreased it (Figure 1B). The total number of migrating cells in all cell lines is presented in Supplementary Figure 2A. Apelin was also shown to modulate the invasiveness of melanoma cells and to induce its metastasis to lymph nodes [32]. Therefore, the effect of apelin on invasion abilities of colon cancer cells was analysed using Transwell™ invasion assay (Figure 1C). Apelin peptides stimulated the invasion of all tested cells trough Matrigel™, imitating the invasion through ECM, whereas ML221 showed a decrease ability to invade. The total number of invading cells of all cell lines was shown in Supplementary Figure 2B.

### 3.2. Apelin Stimulates Blebs Formation in Colon Cancer Cells

**Figure 2:**
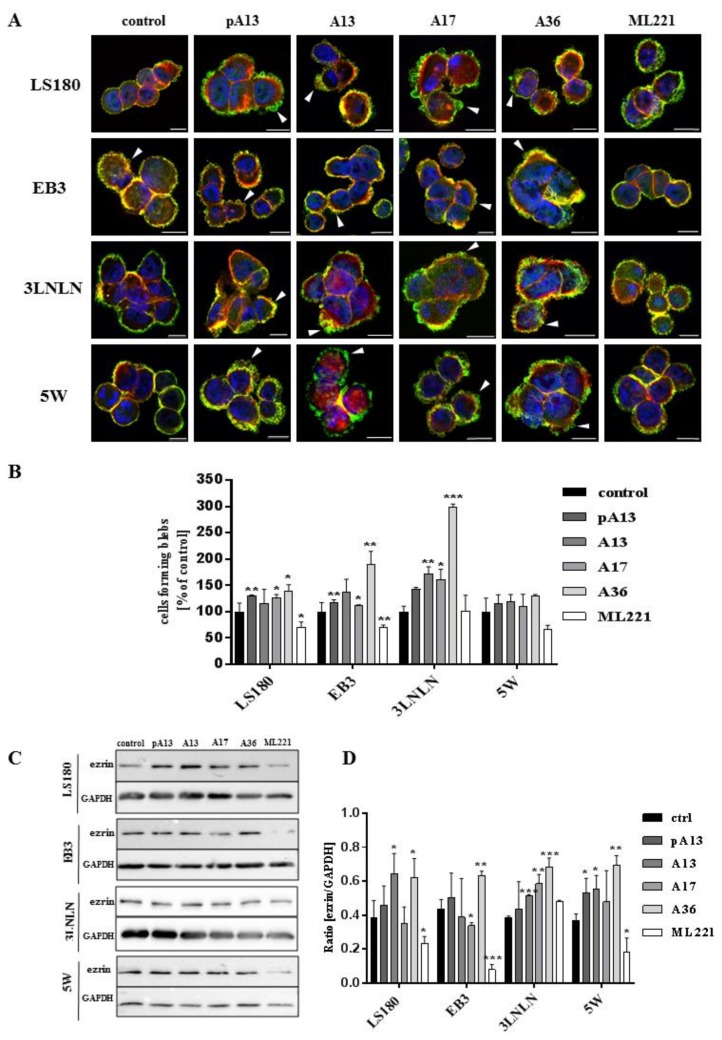
Influence of apelin on blebs formation. (**A**) Representative localization of ezrin (green), filamentous actin (red) and nuclei (blue) in colon cancer cells after apelin peptides (100 nM) or ML221 (100 nM) stimulation. Arrowheads indicate migratory protrusions. Scale bars represent 10 μm; (**B**) Quantification of the number of cells forming blebs after apelin peptides or ML221 treatment, shown as a percent of control cells. Results are expressed as the mean (cells forming blebs) ± SD of three independent experiments. *p* ≤ 0.05 (*), *p* ≤ 0.01 (**), *p* ≤ 0.001 (***). Representative Western blot; (**C**) and densitometric (**D**) analysis for level of ezrin in tested cells incubated with apelin peptides or ML221. The GAPDH level was shown as a loading control. Results are expressed as the mean ± SD of three independent experiments. *p* ≤ 0.05 (*), *p* ≤ 0.01 (**), *p* ≤ 0.001 (***).

All examined cells were characterized by rounded morphology and specific spherical migratory protrusions formation. There are several publications presenting the connection between blebbing and the migration potential of cells [33,34]. These protrusions—called blebs—are typical of cells using ameboid type movement [35]. Moreover, to evaluate if apelin peptides could influence blebs formation, ezrin, a marker of blebs protrusions and filamentous actin were stained using immunocytochemistry (Figure 2A). Fluorescent staining showed increased number of cells forming blebs after apelin peptides stimulation. To confirm this result, cells forming blebs were counted (Figure 2B). Apelin peptides treatment increased the number of cells creating protrusions in LS180, EB3 and 3LNLN cell lines. In LS180 and EB3 cells, ML221 acted in the opposite way by decreasing the number of cells. Additionally, we visualized the effect of apelin on blebs formation in viable cells. For this reason, parental LS180 cells were transfected with LiveAct^®^ plasmid enabling the visualization of filamentous actin as red fluorescence, therefore highlighting the cytoskeleton structure of the cells, including migratory protrusions. The incubation of tested cells with apelin (representative for apelin-36) resulted in increased blebs formation (Supplementary Movie 1B), whereas stimulation with ML221 was characterized by the complete blockage of protrusions creation (Supplementary Movie 1C) in comparison with non-treated cells (Supplementary Movie 1A). Moreover, to verify the differences in ezrin levels observed during fluorescent staining, the level of this protein was analysed using Western blotting (Figure 2C). It came across as apelin elevated the level of ezrin, and APJ antagonist ML221 decreased it, in all cell lines tested, excluding 3LNLN cell line. These findings were confirmed by densitometric analysis (Figure 2D).

Blebs formation is often connected with apoptosis [36]. According to many studies apelin may prevent apoptosis [37,38]; therefore, we examined if apelin could prevent staurosporin (STS)-induced apoptosis of colon cancer cells using Annexin V-FITC and propidium iodide assay (Supplementary Figure 3). Our results showed no ability of apelin to prevent STS-induced apoptosis, as well as no pro-apoptotic effect due to the fact that the apoptotic process has not been enhanced by apelin.

### 3.3. Apelin Influences Actin Polymerisation State and the Level of Cofilin

**Figure 3:**
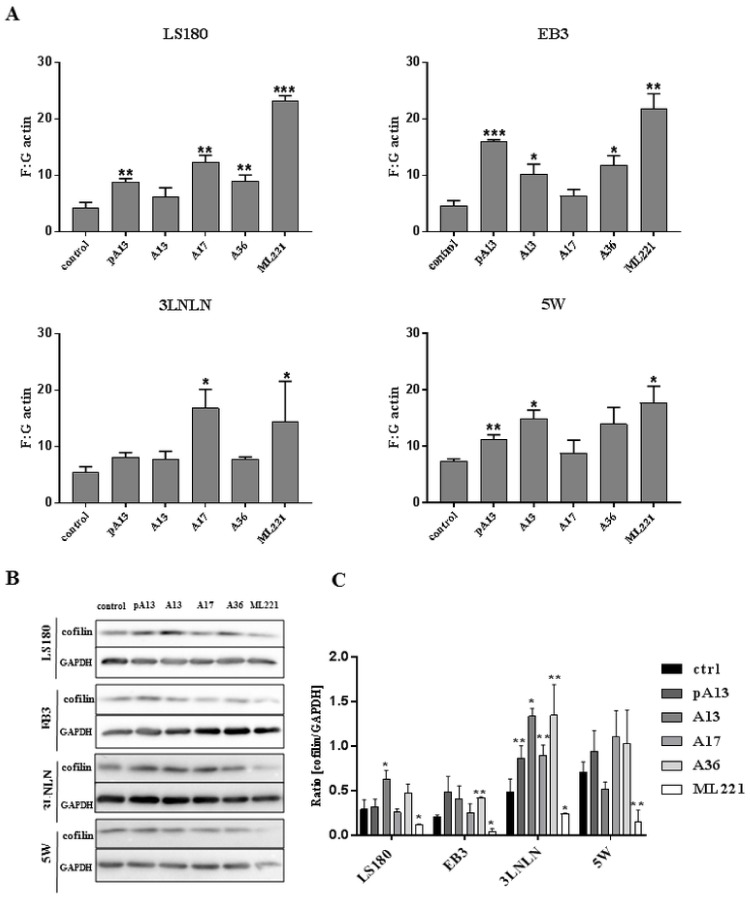
Effect of apelin on actin polymerisation and level of cofilin. (**A**) Ratio of F-actin to G-actin in colon cancer cells after apelin peptides (100 nM) or ML221 (100 nM) treatment. Results are expressed as the mean (F:G ratio) ± SD of three independent experiments. *p* ≤ 0.05 (*), *p* ≤ 0.01 (**), *p* ≤ 0.001 (***). Representative Western blot (**B**) and densitometric (**C**) analysis of levels of cofilin in tested cells incubated with apelin peptides or ML221. The GAPDH level is shown as a loading control. Results are expressed as the mean ± SD of three independent experiments. *p* ≤ 0.05 (*), *p* ≤ 0.01 (**), *p* ≤ 0.001 (***).

One of the major steps in cell movement is migratory protrusions formation. This process is based on actin rearrangement involving actin polymerisation [34]. Therefore, the polymerisation state of cytoplasmic actin was examined in LS180 and sublines. Apelin peptides treatment increased the ratio of filamentous (F-actin) to monomeric (G-actin) actin in all cytoplasmic fractions of colon cancer cells used (Figure 3A). This result suggests that increased levels of filamentous actin after apelin stimulation could be connected with increased migration abilities. However, an increased F:G actin ratio after ML221 treatment was also noticed. Elevated levels of filamentous actin could be associated with altered level of some actin-binding proteins involved in cytoskeleton reorganisation. Cofilin is a multifunctional protein regulating actin dynamics [39]. Decreased levels of cofilin after ML221 treatment were observed in all tested cells (Figure 3B). These findings were confirmed by densitometric analysis (Figure 3C).

### 3.4. Apelin Effects the Proteolytic Abilities of Colon Cancer Cells

**Figure 4:**
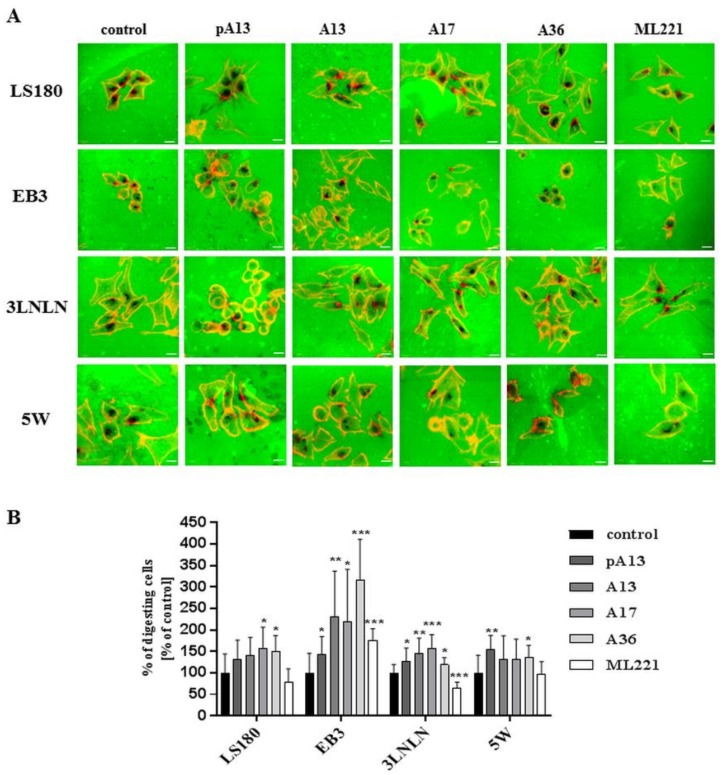
Influence of apelin on proteolytic activity of colon cancer cells. (**A**) The proteolytic activity of colon cancer cells (red) after apelin peptides (100 nM) or ML221 (100 nM) stimulation using FITC-conjugated gelatine (green). Scale bars represent 10 μm; (**B**) The number of cells digesting gelatine was expressed as a percent of digesting cells and results are shown as a percent of control. Results are expressed as the mean (percent of digesting cells) ± SD of three independent experiments. *p* ≤ 0.05 (*), *p* ≤ 0.01 (**), *p* ≤ 0.001 (***).

Invasiveness of cancer cells is usually connected with their ability to degrade the extracellular matrix by proteolytic enzymes [20]. Therefore, the influence of apelin peptides and ML221 on the proteolytic activity of colon cancer cells was examined using gelatine-FITC degradation assay (Figure 4A). FITC-conjugated gelatine presented as a green background. Black dots reflect areas digested by colon cancer cells’ proteases. Stimulation with apelin peptides resulted in an increased number of black areas in all cell lines. Next, digested areas reflecting proteolytic activity of cells were counted (Figure 4B). Our data indicated that apelin peptides elevated proteolytic activity in all cell lines shown as a percent of cells digesting gelatine.

**Figure 5:**
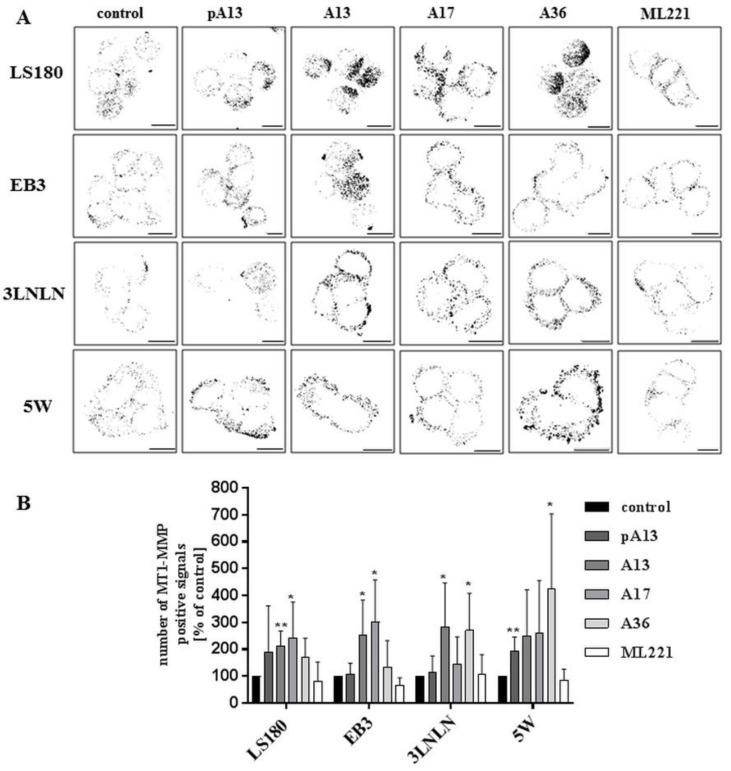
Effect of apelin on the level of MT1-MMP in colon cancer cells. (**A**) The localization of MT1-MMP (black) in tested cells treated with apelin peptides (100 nM) or ML221 (100 nM). Scale bars represent 10 μm; (**B**) Quantification of the number of MT1-MMP positive signals after apelin peptides or ML221 treatment showed as a percent of control cells. Results are expressed as the mean (number of MT1-MMP positive signals) ± SD of three independent experiments. *p* ≤ 0.05 (*), *p* ≤ 0.01 (**).

Metalloproteases are one of the main groups of enzymes involved in degradation and remodelling of ECM, promoting invasion and metastasis [40]. Previous studies showed that apelin could induce the expression of matrix metalloproteases in gastric cancer cells [21]. Therefore, the influence of apelin peptides on the level of MT1-MMP, which belongs to the family of membrane-anchored MMPs, was determined using immunocytochemistry [41]. Apelin stimulation resulted in slightly increased levels of MT1-MMP (Figure 5A), which was confirmed by quantification of the number of MT1-MMP positive signals (Figure 5B).

## 4. Discussion

Many studies have indicated that adipose tissue could be involved in the development of obesity-related disorders. Fat storage is not the only function of adipose tissue; it also secrets biologically active compounds regulating metabolic homeostasis, such as adipokines [42]. These adipose tissue-derived peptides regulate several physiological processes, including energy balance, appetite regulation, insulin sensitization, inflammatory response and vascular homeostasis [43]. Thus, under obesity, the increased secretion of adipokines leads to metabolic disturbances that could play role in further disorder development, including type 2 diabetes and cardiovascular diseases [42]. The correlation between carcinogenesis and increased body mass index suggests that obesity plays an important role in colorectal carcinogenesis [3].

Apelin, which belongs to the adipokines family, also plays a role in obesity. In a group of obese female children, apelin-12 plasma levels were significantly increased in comparison with non-obese patients [44]. Moreover, in obese men with colon cancer, the level of plasma apelin-12 was also increased [45]. Nevertheless, tumour-associated apelin, instead of serum apelin levels, is closely connected with more advanced clinical features, including tumour differentiation and distant metastases in the case of gastric cancer [21]. Moreover, apelin peptides increased migration and invasion abilities of several types of cancer, including lung adenocarcinoma and gastric cancer [19,21,22]. However, the mechanism of apelin’s action in colon cancer is still unknown.

In our study, we examined the influence of apelin peptides on colon cancer cell motility. To achieve this goal, we used four cell lines with different migratory potential: parental LS180 colon cancer cell line, selected in vitro from parental cell line EB3 and selected in vivo from EB3, 3LNLN and 5W cell lines, that metastasized to the lymph nodes and to the liver, respectively [25]. Various apelin peptides have been used in many studies; however, controversies have emerged concerning the activity or type of these peptides. Due to this fact, we examined and compared four apelin peptides: [Pyr1] apelin-13, apelin-13, -17 and -36. All these peptides are present in human serum and are able to activate the apelin receptor APJ [46]. As an additional control, we used APJ functional antagonist ML221, which binds to the receptor and blocks the possibility of linking apelin and activating APJ [47]. Based on bibliographical data [12] and our results, we selected 100 nM concentrations of all apelin peptides and as well as ML221, which has no cytotoxic effect on in vitro cell cultures, and are able to influence cellular processes. First, we examined the effect of apelin peptides on migration and invasion abilities. Migration assay revealed, that all apelin peptides stimulate migration in all cell lines. Similar results were obtained when examining apelin-13 influence on lung adenocarcinoma A549 migration abilities. Apelin also contributed to lung adenocarcinoma resistance to chemotherapy-induced inhibition of cell metastasis [19]. Therefore, we checked the invasion facilities of tested cells after apelin treatment. In this case cells were seeded into Matrigel™-coated Transwell™ inserts, to imitate the invasion of cancer cells through ECM. These results resembled those obtained with the migration test. The same findings were obtained by Feng et al., who observed that apelin increased the invasion rate of gastric cancer cells by upregulation of several cytokines known to facilitate tumour progression, such as BMP-2, IL1 and IL6. Nevertheless the type of apelin was not specified [21].

Migration of cells is connected to the creation of migratory protrusions. Typical structures in cells characterized with a rounded morphology are blebs. These actin-rich structures can occur anywhere on the plasma membrane [48]. Several publications have presented the connection between blebbing and the migration potential of cells [33,34]. The experimental induction of blebs formation in non-invasive cells resulted in increased ability of these cells to invade into 3D matrices. Moreover, bleb-associated cell motility is connected with multiple molecular mechanisms including actin-mediated bleb retraction [49]. Since the examined colon cancer cells are able to form blebs protrusions [35], we investigated whether apelin stimulation influenced their formation. We used immunocytochemistry to visualise ezrin and filamentous actin. Ezrin belongs to the ABPs and is an actin-membrane linker, essential for several cellular processes, including cell shape determination, adhesion and motility [50]. It localizes in migratory protrusions [34]. Therefore, we used ezrin as a marker of blebs. Our observations suggest, that apelin peptides increased the number of cells forming blebs. These implications were confirmed (except in the 5W cell line) by quantifying the number of blebbing cells. We also visualized filamentous actin in viable parental colon cancer cells. The movie shows the dynamic process of protrusions creation after apelin treatment. Moreover, the differential number of cells forming blebs caused by apelin stimulation resulted in altered ezrin levels. All these findings indicate that apelin peptides may increase migration abilities by affecting protrusion activity.

Migratory protrusion formation is usually connected with actin cytoskeleton rearrangement and recruitment of actin-binding proteins [34]. Moreover, increased migration abilities of colon cancer cells are connected with a larger F- to G-actin ratio [24]. For this reason, we examined the actin polymerisation state in the cytoplasmic fraction of colon cancer cells incubated with apelin. We focused on test based upon simple enzyme-inhibitor reaction. The specific feature of G-actin to inhibit DNAse I allows us to quantitatively count the ratio of F-actin to G-actin and statistically elaborate the influence of apelin on this parameter. Moreover, the ratio of F:G actin was examined in cytosol—the most dynamic fraction of cell, what was important for examining the dynamic reorganisation of the actin cytoskeleton after apelin treatment. In colon cancer cells apelin peptides increased F:G actin ratio, which suggests that apelin shifted the actin balance onto actin polymerisation in tested cells. These findings are consistent with previous results; intense blebs formation after apelin treatment is correlated with actin polymerisation. However, even more intense effect on F:G actin ratio was observed after APJ antagonist stimulation. To explain these observations, we checked the level of cofilin. This actin-binding protein is involved in actin cytoskeleton rearrangement [51]. The connection with apelin-13 and cofilin was previously revealed by Lv et al. In human lung adenocarcinoma cells, this peptide induced cell migration through PAK-1/cofilin-dependent pathway [19]. We observed an increase in the F:G ratio after APJ antagonist treatment, which could be due to disorders in actin organisation caused by the decrease in cofilin level. A similar effect was observed by Popow-Woźniak et al., who examined the influence of constitutively inactive cofilin on migration and actin dynamics of LS180 cells. Cells expressing constitutively inactive cofilin were characterized by elevated actin polymerization state, as well as decreased ability to migrate. The authors suggested that it could be connected with the inability to depolymerize the actin filament as a result of a reduced amount of monomeric actin in favour of filamentous actin. Perturbations in actin dynamics explain the decreased migration abilities of colon cancer cells [52].

Invasion of cancer cells is tightly connected with its proteolytic abilities. Cancer cells secrete proteases that degrade ECM, clearing the way for the metastasis [53]. Therefore, we checked the effect of apelin on proteolytic enzyme activity in colon cancer cells. For this purpose, we decided to use FITC-conjugated gelatine assay. Our results suggested that apelin increased the number of cells degrading gelatine, which was also confirmed quantitatively. One of the group of enzymes, that play a key role in metastasis by degrading ECM is the metalloproteases. MT1-MMP (MMP-14) may degrade the ECM composed of different components, including collagens type-I, -II and –III, fibronectin, vitronectin, laminin-1, fibrin, aggrecan, versican, perlecan, casein, nidogen, serpins and tenascin-C [53,54,55]. Only four members of the MMPs (MMP-1, MMP-8, MMP-13 and MT1-MMP) can degrade fibrillar collagens in their triple-helical domain which leave the molecules thermally unstable so that they unwind to form gelatine after which they can be degraded by other members of the MMP family. However, MT1-MMP is also able to degrade gelatine [56]. Due to the fact that we observed the effect of apelin treatment on proteolytic activities in FITC-conjugated gelatine assay and in gastric cancer cell lines where apelin treatment stimulated the protein level of MMPs [21], we visualised MT1-MMP-membrane-anchored MMP using immunocytochemistry. This MMP localised as small dots on the cell membrane surface, thus we were able to count the number of fluorescent spots. Our findings revealed that apelin peptides increased the number of MT1-MMP positive signals. All these data suggest that apelin peptides increase colon cancer cell migration through stimulation of proteolytic enzyme activity.

APJ antagonist ML221, used in our study as an additional control, revealed interesting findings. Incubation of colon cancer cells with APJ antagonist resulted in decreased migration and invasion, as well as a reduced number of cells forming blebs. This opposite effect in comparison to apelin treatment indicated that inhibition of apelin receptor and its downstream signalling may affect the capability of cancer cells to migrate. In line with our results it is a small evidence that ML221 decreased migration and invasion abilities of cholangiocarcinoma cell lines. Moreover, it also reduced tumour growth in vivo [54]. The opposite effect of ML221 obtained during migration and invasion assay was not observed pending proteolytic activity examination.

Many studies and our research indicate that apelin peptides influence some cellular processes, such as proliferation, migration and invasion. However, the mechanism of its action is still unknown. It could be the result of the presence of more than one apelin receptor. The most known and established apelin receptor is APJ, which belongs to the G protein-coupled receptor family [6]. This huge family includes more than 700 proteins, including 200 orphan receptors [55]. All of them are 7-transmembrane proteins sharing several structural features. Since Tatemoto et al. found apelin as a ligand for orphan APLNR [4], several mechanisms including signalling pathways were examined [7]. The latest data indicated that the orphan G protein-coupled receptor 25 also could be activated by apelin in non-mammalian vertebrates [56]. This new finding suggests that orphan receptors could be still potentially activated by apelin. Interestingly, APJ antagonist ML221 could also inhibit other receptors, including kappa opioid or the benzodiazepinone receptors [47]. Thereupon, the data are still inconsistent and controversial and it could be difficult to examine mechanism of apelin’s action. As such, further studies are needed.

## 5. Conclusions

In summary, our studies indicate that apelin increases the migration and invasion abilities of colon cancer cells by several possible mechanisms. Here, for the first time, we examined and compared a panel of four different apelin peptides in all experiments. All tested peptides displayed similar activity, which can be connected with sharing the same sequence on the C-end of their structure. Overall, apelin stimulated migratory protrusions formation, and affected actin cytoskeleton rearrangement and the proteolytic abilities of examined cells. These findings indicate that there is a connection between apelin and carcinogenesis and cancer spreading. The influence of tumour environment, including adipose tissue secreting apelin, on colon cancer development could be an interesting future research area and a promising target for the establishment of novel anti-cancer therapy.

## Supplementary Materials

The supplementary materials are available online.
