# Supplementary figures and images for: Apelin Effects Migration and Invasion Abilities of Colon Cancer Cells
Source: Cells. 2018 Aug 20;7(8):113. doi: 10.3390/cells7080113 (PMC6115746; doi:10.3390/cells7080113)

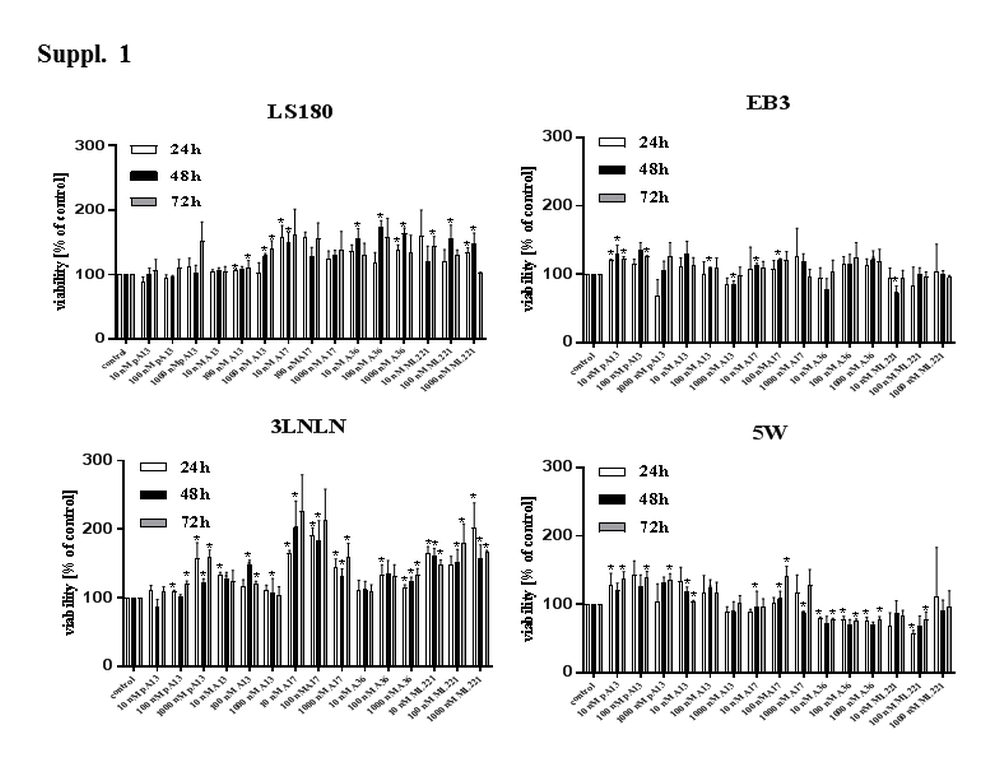

Supplement: Supplementary file 1 [file cells-07-00113-s001.zip › Supplementary figures/Supplementary Figure 1..tif]

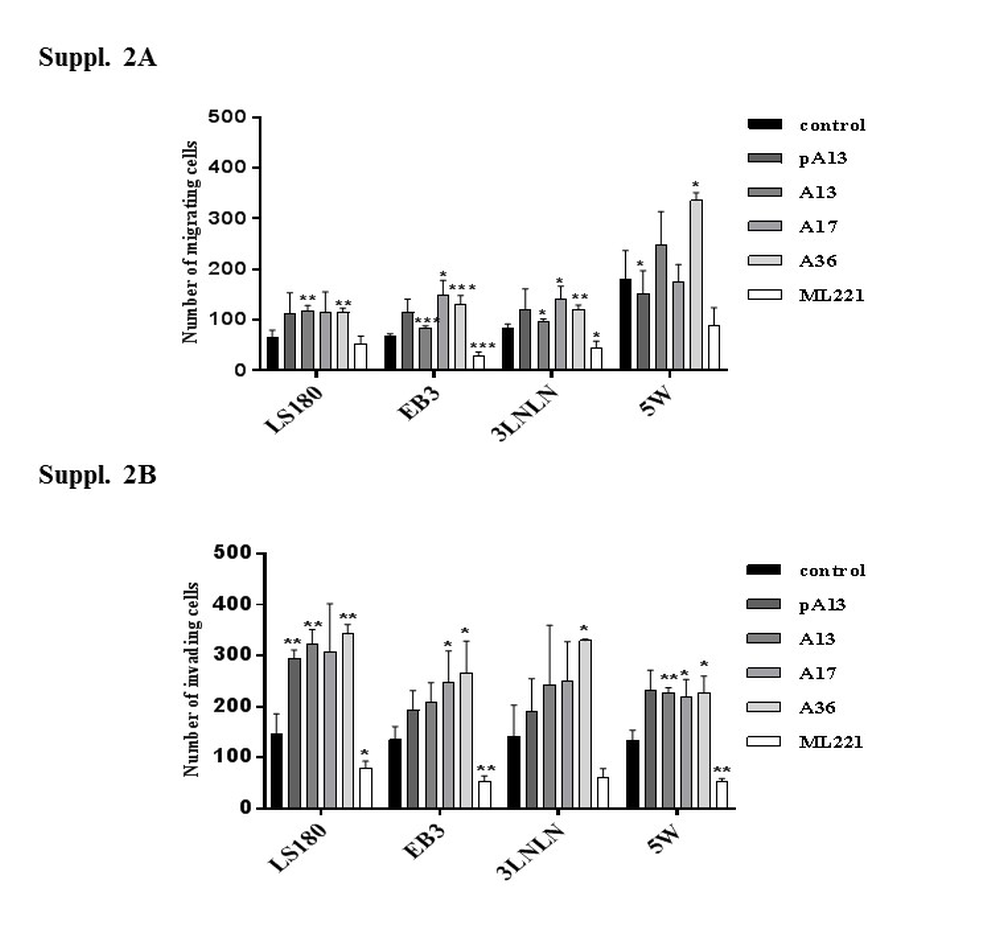

Supplement: Supplementary file 1 [file cells-07-00113-s001.zip › Supplementary figures/Supplementary Figure 2..tif]

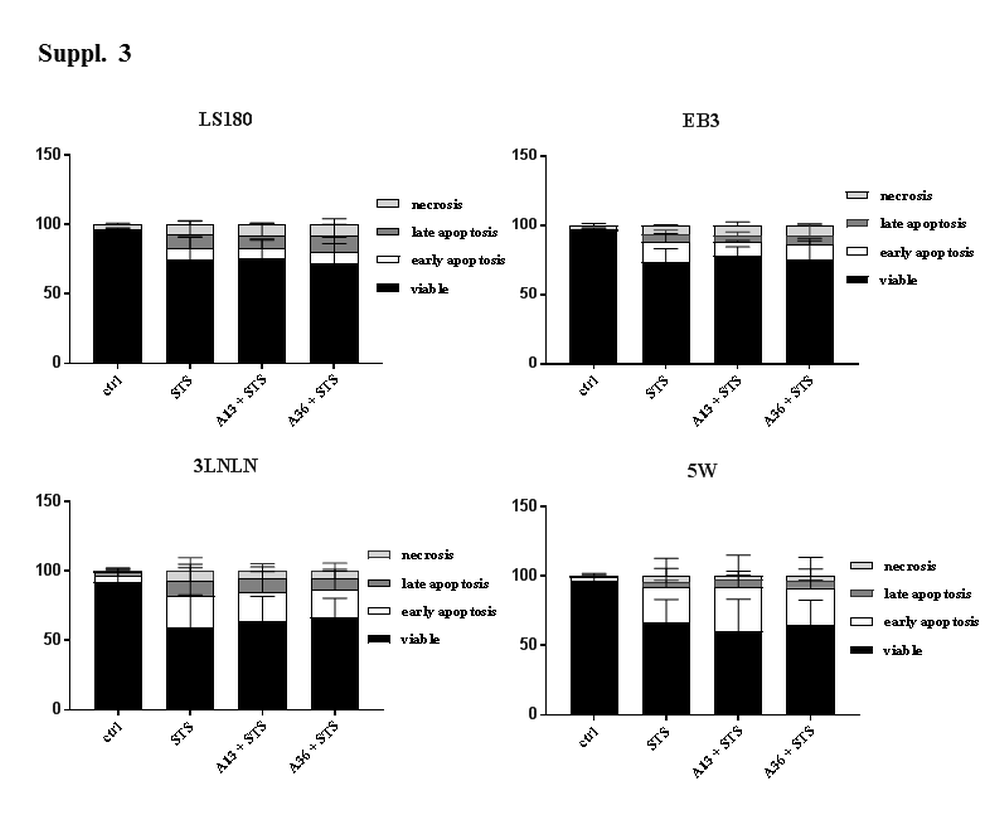

Supplement: Supplementary file 1 [file cells-07-00113-s001.zip › Supplementary figures/Supplementary Figure 3..tif]
